# Leveraging Innovative Electronic Health Record Data to Characterize Social Determinants of Health Among Survivors of Cancer in Persistent Poverty Areas: Cross-Sectional Study

**DOI:** 10.2196/81054

**Published:** 2026-04-13

**Authors:** Shiori Tanaka, Laura Q Rogers, Lisa Zubkoff, Lori Bateman, Wendy Demark-Wahnefried, Gabrielle Rocque, Sejong Bae, Maria Pisu, James Booth, Carrie R Howell

**Affiliations:** 1 Department of Medicine, University of Alabama at Birmingham, 1717 11th Ave South, Birmingham, AL, 35294, United States, 1 2059345685; 2 Department of Nutrition Sciences, University of Alabama at Birmingham, Webb 518, 1720 2nd Avenue South, Birmingham, AL, 35294, United States

**Keywords:** social determinants of health, survivors of cancer, persistent poverty, electronic health records, adapted interventions

## Abstract

**Background:**

Residents in persistent poverty areas experience higher cancer mortality due to social determinants of health that negatively affect multiple factors, including health behaviors.

**Objective:**

This study aimed to characterize demographic, clinical, and social determinant of health factors among survivors of cancer in persistent poverty areas using electronic health record (EHR) data—including an embedded social risk screener and natural language processing (NLP) of social work notes—to inform community-engaged adaptation of lifestyle interventions.

**Methods:**

EHR data from a large multispecialty group practice were extracted for patients with cancer residing in zip codes inclusive of persistent poverty areas targeted for a health behavior intervention and receiving care between January 2018 and November 2023. Self-reported social determinant of health data were obtained using the Protocol for Responding to and Assessing Patients’ Assets, Risks, and Experiences (PRAPARE) and through NLP of social histories from a social work visit.

**Results:**

We identified 2672 unique patients with cancer, of whom 578 (21.6%) had PRAPARE data and 1597 (59.8%) had social history data available for analysis. The most common cancers among survivors (n=1420, 53.1% female; n=1299, 48.6% Black individuals; mean age 65.2, SD 13.7 years) included breast (n=536, 20.1%), prostate (n=400, 15%), and lymphoid or hematopoietic (n=323, 12.1%) cancer. Among survivors in persistent poverty areas (n=509, 19%; all with a high Social Vulnerability Index), 34.6% (176/509) were single, 55.4% (282/509) had Medicare coverage (with only 73/509, 14.3% having private insurance), 36.5% (186/509) had obesity, 63.9% (325/509) had hypertension, and 31.2% (159/509) had diabetes. Of survivors in persistent poverty areas with PRAPARE data, 15.8% (19/120) lacked transportation, 4.2% (5/120) lived with housing insecurity, and 6.7% (8/120) felt unsafe where they lived.

**Conclusions:**

Innovative EHR and NLP approaches identified several socioeconomic and safety-related challenges along with opportunities for health behavior interventions to leverage Medicare coverage and target multiple comorbidities when adapting interventions for survivors of cancer living in persistent poverty areas.

## Introduction

Persistent poverty areas, defined by the US Census Bureau, are counties or census tracts where at least 20% of the population has lived below the federal poverty level for 30 or more years. Despite medical advancements improving cancer survival [1], mortality rates remain disproportionately high in these areas [2]. Community-based research is imperative for adapting interventions to this population, ensuring effectiveness and reducing disparities [3].

Dietary and physical activity interventions help prevent cancer recurrence and mitigate comorbidities such as obesity and diabetes [4-6]. However, implementing these interventions in persistent poverty areas is challenging, with ongoing uncertainties regarding their acceptability, feasibility, and sustainability. These areas experience adverse social determinants of health (SDOH), including food insecurity, transportation barriers, and financial strains, all of which limit engagement in interventions [7]. However, these social risk data have not been systematically collected or integrated into clinical records, limiting their availability for tailoring intervention strategies [3].

Despite increasing use of electronic health records (EHRs) to improve health care quality, they remain underused for contextualizing cancer control efforts in persistent poverty areas. EHR data offer a unique opportunity to systematically characterize the demographic, clinical, and social risk profiles of local populations, providing actionable information for adapting interventions to community-specific needs. To date, only 1 study has used EHR data to characterize survivors of cancer in persistent poverty areas for future community interventions [8], but it did not capture key SDOH (eg, food insecurity) or integrate the findings into a community-engaged co-design process. With growing interest in integrating SDOH and social risk screening into health systems [9,10], richer data are becoming more readily available to characterize local populations of interest and design targeted interventions.

The Leveraging Adaptation and Multilevel Implementation Strategies to Address Unique Health Promotion Challenges Among Cancer Survivors in Persistent Poverty Areas (LEAP) study (U54CA280770) seeks to adapt evidence-based dietary and physical activity interventions in persistent poverty areas while building community capacity for implementation and sustainability. This report describes an innovative use of EHR data, including social risk data, to identify demographic, medical, and SDOH factors potentially relevant to diet and exercise intervention adaptation and delivery for survivors and cosurvivors of cancer living in persistent poverty areas. The objective of this descriptive study was to leverage EHR data—including an embedded social risk screener and natural language processing (NLP) of social work notes—to characterize demographic, clinical, and SDOH factors among survivors of cancer residing in persistent poverty areas and translate these findings into information for community-engaged adaptation of lifestyle interventions.

## Methods

### Population

**Figure 1.**
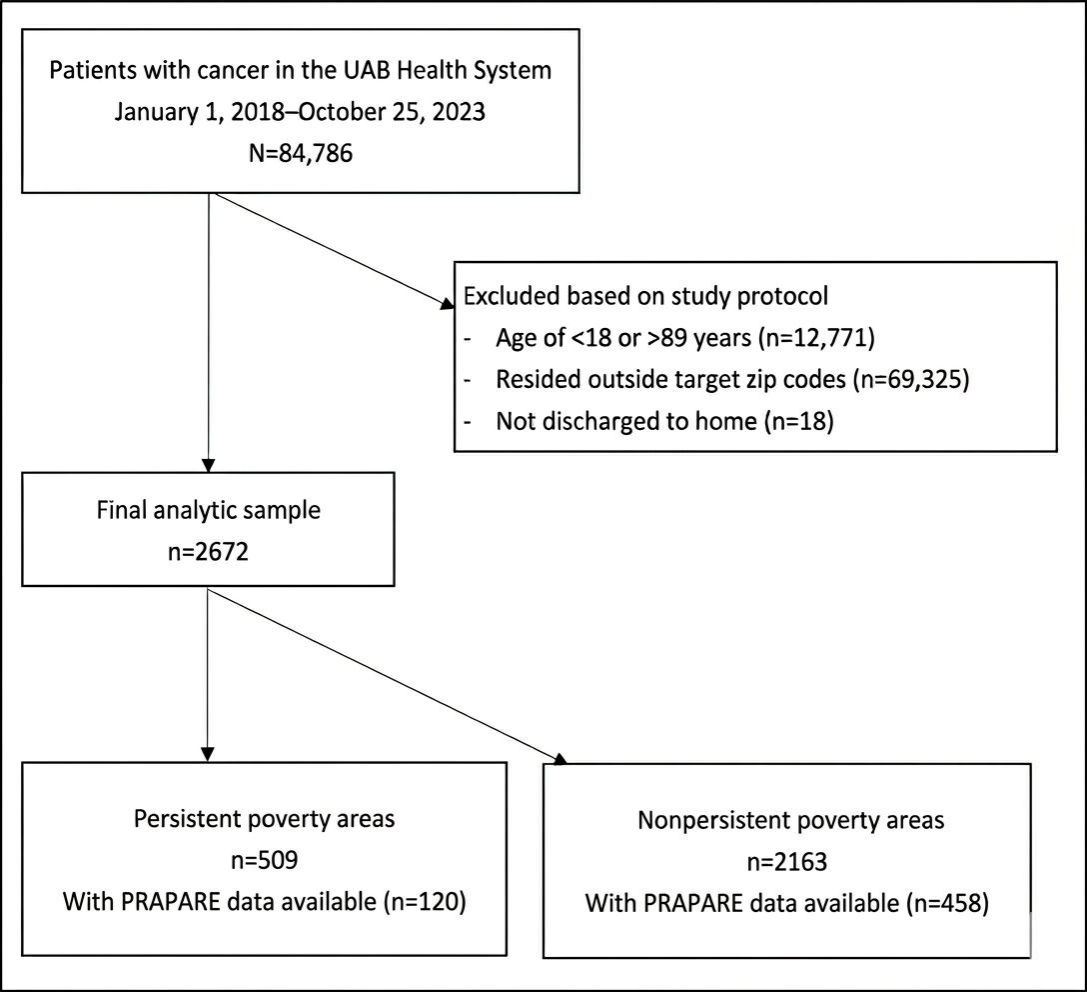
Study flow diagram. PRAPARE: Protocol for Responding to and Assessing Patients’ Assets, Risks, and Experiences; UAB: University of Alabama at Birmingham.

We extracted EHR data from the University of Alabama at Birmingham Health System, a multispecialty group practice. The study population included patients with cancer who (1) received medical services between January 2018 and November 2023, (2) were aged 18 to 89 years, (3) were considered potential intervention participants (ie, returned home), and (4) resided in predefined communities (per the study protocol) within the Birmingham metropolitan area of Alabama that were planned locations for LEAP study adaptation and implementation (Figure 1).

Because the health system’s data extraction is operated by zip code, patients were initially identified using zip codes overlapping with target persistent poverty census tracts; as a result, the extracted population included residents of both persistent and nonpersistent poverty areas. Patient addresses were then geocoded to census tracts using institutional geocoding services. Persistent poverty status was determined by linking census tract codes to US Census Bureau persistent poverty designations. The Social Vulnerability Index (SVI) was linked at the census tract level using Centers for Disease Control and Prevention and Agency for Toxic Substances and Disease Registry SVI data. Consistent with the National Cancer Institute definition, survivors of cancer were defined as individuals with a documented cancer diagnosis from the time of diagnosis onward regardless of current treatment status. This definition was operationalized by identifying patients with cancer-related *International Classification of Diseases, Ninth Revision*; *International Classification of Diseases, Tenth Revision*; or Systematized Nomenclature of Medicine–Clinical Terms diagnostic codes in their EHR.

EHR data provided clinical information, including cancer type, body weight status, and comorbid conditions, as well as demographic characteristics (eg, age and race or ethnicity) and SDOH (eg, insurance and marital status). Census tract codes were used to identify persistent poverty areas among those living in low-income zip code geographic areas and were used to link the SVI, a composite neighborhood-level index assessing social vulnerabilities [11]. Additional SDOH data were available through the Protocol for Responding to and Assessing Patients’ Assets, Risks, and Experiences (PRAPARE) [12], a patient-reported social risk screener integrated into the EHR system at our institution in the Social Work unit in 2020 [13]. PRAPARE includes 21 questions across 4 domains: personal characteristics, family and home, money and resources, and social and emotional health [12].

Social history notes collected via social work visits were organized in the EHR using Social Interventions Research and Evaluation Network (SIREN) [14] nomenclature. These data were analyzed using NLP to identify key SDOH themes aligned with the Centers for Medicare and Medicaid Services [15]. We applied a rule-based NLP approach to identify events related to unmet needs from both structured questionnaire items and free-text patient responses recorded in the EHR. Text data were first preprocessed through tokenization and removal of stop words, punctuation, and noninformative characters, followed by normalization (eg, lowercasing and stemming) to standardize text across sources [16,17]. This preprocessing was applied consistently to both questionnaire prompts and patient responses.

We then conducted keyword-based text matching to identify SDOH-related events across 5 predefined domains: food insecurity, housing instability, transportation needs, interpersonal safety, and utility difficulties. The domain-specific keywords and phrases used to extract relevant events and identify unmet needs or negative responses are detailed in Table S1 in Multimedia Appendix 1. For each SDOH domain, patients were classified as experiencing unmet needs or insecurity if their most recent response indicated an adverse condition. Patients without such indicators were classified as having no identified unmet need (ie, secure) for that domain.

We summarized findings in tables for community engagement processes (eg, informing co-design and focus group guides) and intervention adaptation (eg, educational content topics, potential delivery channels, and anticipated challenges). Although hypothesis testing was not our primary focus, 2-tailed *t* tests, and chi-square or Fisher exact tests were used to estimate differences between survivors living in persistent poverty areas and those not living in these areas to examine trends in the data. Distributions of continuous variables (age and BMI) were approximately symmetric, supporting the use of *t* tests for group comparisons. Given the descriptive nature of this study and the absence of a priori hypotheses, *P* values are presented to identify potential trends rather than to establish statistical significance. No adjustment for multiple comparisons was applied, and the results should be interpreted as exploratory. All analyses were conducted using R (version 4.3.2; R Foundation for Statistical Computing).

### Ethical Considerations

This study was approved by the Institutional Review Board at UAB (approval number IRB-300010921), with informed consent waived. This study was conducted in accordance with the study protocol. Only patients who met the predefined inclusion criteria were included. To protect participant privacy, we avoided reporting overly granular or potentially identifiable information in the results, including zip codes and census tracts. No compensation was provided to the participants.

## Results

Among 2672 survivors, the mean age was 65.2 (SD 13.7) years, 1420 (53.1%) were female, and 1299 (48.6%) were Black individuals (Table 1). Common cancers included breast (n=536, 20.1%), prostate (n=400, 15%), and lymphoid or hematopoietic (n=323, 12.1%) cancer. A total of 19% (n=509) of the survivors lived in persistent poverty areas, with high obesity (186/509, 36.5%) and hypertension (325/509, 63.9%). These survivors were more likely to be Black individuals (297/509, 58.3% vs 1002/2163, 46.3% in nonpersistent poverty areas), insured by Medicaid (64/509, 12.6% vs 145/2163, 6.7% in nonpersistent poverty areas) or uninsured (35/509, 6.9% vs 95/2163, 4.4% in nonpersistent poverty areas), single (176/509, 34.6% vs 576/2163, 26.6% in nonpersistent poverty areas), and from neighborhoods with higher SVI (509/509, 100% vs 940/2163, 43.5% in nonpersistent poverty areas).

**Table 1.** Characteristics of survivors of cancer within a large multispecialty group practice (overall and by whether the survivor lived in a persistent poverty census tract).

Variable	Overall (N=2672)	Residing in persistent poverty areas^a^ (n=509)	Residing in nonpersistent poverty areas (n=2163)	*P* value^b^
Demographics				
Age at assessment (years), mean (SD)	65.2 (13.7)	65.4 (12.8)	65.2 (13.9)	.72
Sex, n (%)				.23
Female	1420 (53.1)	259 (50.9)	1161 (53.7)	
Male	1252 (46.9)	250 (49.1)	1002 (46.3)	
Race or ethnicity, n (%)				<.001
Asian	290 (10.9)	101 (19.8)	189 (8.7)	
Black	1299 (48.6)	297 (58.3)	1002 (46.3)	
Hispanic	42 (1.6)	19 (3.7)	23 (1.1)	
White	959 (35.9)	76 (14.9)	883 (40.8)	
Other^c^	82 (3.1)	16 (3.1)	66 (3.1)	
Spoken language, n (%)				<.001
English	2467 (92.3)	445 (87.4)	2022 (93.5)	
Other or unknown	205 (7.7)	64 (12.6)	141 (6.5)	
Health insurance, n (%)				<.001
Private	645 (24.1)	73 (14.3)	572 (26.4)	
Medicaid	209 (7.8)	64 (12.6)	145 (6.7)	
Medicare	1460 (54.6)	282 (55.4)	1178 (54.5)	
Uninsured	130 (4.9)	35 (6.9)	95 (4.4)	
Other^c^	228 (8.5)	55 (10.8)	173 (8.0)	
Marital status, n (%)				<.001
Married or with a life partner	1013 (37.9)	136 (26.7)	877 (40.5)	
Single	752 (28.1)	176 (34.6)	576 (26.6)	
Other^c^	907 (33.9)	197 (38.7)	710 (32.8)	
Clinical measures				
BMI (kg/m^2^)^d^, mean (SD)	29.4 (9.51)	29.1 (9.00)	29.5 (9.63)	.39
BMI category (kg/m^2^), n (%)				.77
<25	791 (29.6)	153 (30.1)	638 (29.5)	
25 to <30	705 (26.4)	127 (25.0)	578 (26.7)	
≥30	1019 (38.1)	186 (36.5)	833 (38.5)	
Comorbidity^e^, n (%)				
Diabetes mellitus	858 (32.1)	159 (31.2)	699 (32.3)	.68
Hypertension	1714 (64.1)	325 (63.9)	1389 (64.2)	.92
Hyperlipidemia	1106 (41.4)	208 (40.9)	897 (41.5)	.84
Coronary artery disease	519 (19.4)	103 (20.2)	416 (19.2)	.65
Overall comorbidity	1877 (70.2)	349 (68.6)	1528 (70.6)	.39
Cancer site^e^, n (%)				.01
Breast	536 (20.1)	108 (21.2)	428 (19.8)	
Prostate	400 (15.0)	87 (17.1)	313 (14.5)	
Lymphoid or hematopoietic	323 (12.1)	37 (7.3)	286 (13.2)	
Lung	151 (5.7)	31 (6.1)	120 (5.5)	
Colorectal	139 (5.2)	30 (5.9)	109 (5.0)	
Skin	81 (3.0)	5 (1.0)	76 (3.5)	
Bones or soft tissue	61 (2.3)	13 (2.6)	48 (2.2)	
Gastrointestinal	218 (8.2)	38 (7.5)	180 (8.3)	
Genitourinary other than prostate	275 (10.3)	65 (12.8)	210 (9.7)	
Head and neck	142 (5.3)	32 (6.3)	110 (5.1)	
Brain or central nervous system	38 (1.4)	4 (0.8)	34 (1.6)	
Other	308 (11.5)	59 (11.6)	249 (11.5)	
Social Vulnerability Index^f^, n (%)				<.001
Low (0 to <0.33)	429 (16.1)	0 (0.0)	429 (19.8)	
Moderate (0.33 to <0.66)	606 (22.7)	0 (0.0)	606 (28.0)	
High (≥0.66)	1449 (54.2)	509 (100.0)	940 (43.5)	

a Persistent poverty areas were defined as census tracts in which 20% or more of the population has lived below the poverty level for at least 30 years.

b Continuous and categorical variables were compared using a *t* test and chi-square test or Fisher exact test, respectively.

c “Other” includes “other,” “multiple,” and “decline to answer” for race and ethnicity; “other” and “missing” for health insurance; and “separated,” “divorced,” “widowed,” and “unknown” for marital status.

d A total of 5.9% (157/2672) of patients had missing data.

e Diagnoses were based on the *International Classification of Diseases, Ninth Revision*; *International Classification of Diseases, Tenth Revision*; or Systematized Nomenclature of Medicine codes.

f The Social Vulnerability Index (SVI) summarizes the extent to which a community is socially vulnerable to disaster. It was developed by the Centers for Disease Control and Prevention. SVI values range from 0 (least vulnerable) to 1 (most vulnerable). Data were missing for 7.0% (188/2672) of the participants, all from nonpersistent poverty areas.

Richer SDOH data were available for 2 subsets of patients: those with PRAPARE data and those with SIREN data. Of the survivors with PRAPARE data available (578/2672, 21.6%), survivors from persistent poverty areas (120/578, 20.8%) reported lack of transportation (19/120, 15.8%), 4.2% (5/120) reported housing insecurity, and 6.7% (8/120) reported feeling unsafe where they lived (Table 2). While no single social risk differed significantly between the 2 groups, survivors in persistent poverty areas reported a higher mean number of social risks when compared with those in nonpersistent poverty areas (5.28, SD 1.84 vs 4.55, SD 1.92). Of those with SIREN data available through NLP (1597/2672, 59.8%), 13.6% (41/302) of survivors in persistent poverty areas experienced food insecurity (Table S2 in Multimedia Appendix 1).

**Table 2.** Prevalence of social determinants of health extracted from the electronic health record (EHR) using Protocol for Responding to and Assessing Patients’ Assets, Risks, and Experiences (PRAPARE) questions: survivors of cancer overall and by residence in persistent poverty areas vs nonpersistent poverty areas^a^

PRAPARE tool responses	Overall (n=578)	Residing in persistent poverty areas^b^ (n=120)	Residing in nonpersistent poverty areas (n=458)	*P* value^c^
Personal characteristics, n (%)				
Migrant work in the last 2 years				>.99
Yes	5 (0.9)	1 (0.8)	4 (0.9)	
No	520 (90.0)	108 (90.0)	412 (90.0)	
Missing or refused to answer	53 (9.2)	11 (9.2)	42 (9.2)	
Veteran status				.07
Yes	42 (7.3)	14 (11.7)	28 (6.1)	
No	477 (82.5)	96 (80.0)	381 (83.2)	
Missing or refused to answer	59 (10.2)	10 (8.3)	49 (10.7)	
Family and home, n (%)				
Housing status				.72
Lack of housing	12 (2.1)	3 (2.5)	9 (2.0)	
Have housing	550 (95.2)	115 (95.8)	435 (95.0)	
Missing or refused to answer	16 (2.8)	2 (1.7)	14 (3.1)	
Housing security				>.99
Worried	24 (4.2)	5 (4.2)	19 (4.1)	
Not worried	523 (90.5)	108 (90.0)	415 (90.6)	
Missing or refused to answer	31 (5.4)	7 (5.8)	24 (5.2)	
Money and resources, n (%)				
Highest educational grade completed				>.99
High school education or lower	262 (45.3)	56 (46.7)	206 (45.0)	
Higher than a high school education	129 (22.3)	27 (22.5)	102 (22.3)	
Missing or refused to answer	187 (32.4)	37 (30.8)	150 (32.8)	
Current work				.09
Unemployed	232 (40.1)	50 (41.7)	182 (39.7)	
Part time or other	26 (4.5)	6 (5.0)	20 (4.4)	
Unemployed but not seeking work^d^	212 (36.7)	49 (40.8)	163 (35.6)	
Full-time work	46 (8.0)	3 (2.5)	43 (9.4)	
Missing or refused to answer	62 (10.7)	12 (10.0)	50 (10.9)	
Income between 100% and 200% of the FPL^e^	26 (4.5)	6 (5.0)	20 (4.4)	
Income more than 200% of the FPL	20 (3.5)	2 (1.7)	18 (3.9)	
Reported resource needs				.93
Medicine or health care needs	82 (14.2)	17 (14.2)	65 (14.2)	
Resource needs^f^	30 (5.2)	6 (5.0)	24 (5.2)	
No needs	408 (70.6)	83 (69.2)	325 (71.0)	
Missing or refused to answer	58 (10.0)	14 (11.7)	44 (9.6)	
Transportation				.16
Lacking transportation	68 (11.8)	19 (15.8)	49 (10.7)	
Having transportation	476 (82.4)	94 (78.3)	382 (83.4)	
Missing or refused to answer	34 (5.9)	7 (5.8)	27 (5.9)	
Social and emotional health, n (%)				
Social integration and support				.32
≤5 times a week	275 (47.6)	61 (50.8)	214 (46.7)	
>5 times a week	220 (38.1)	40 (33.3)	180 (39.3)	
Missing or refused to answer	83 (14.4)	19 (15.8)	64 (14.0)	
Stress level rating				.62
High and medium-high stress^g^	198 (34.3)	39 (32.5)	159 (34.7)	
Less stress	291 (50.3)	64 (53.3)	227 (49.6)	
Missing or refused to answer	89 (15.4)	17 (14.2)	72 (15.7)	
Feeling safe where they lived				.10
No or unsure	21 (3.6)	8 (6.7)	13 (2.8)	
Yes	429 (74.2)	89 (74.2)	340 (74.2)	
Missing or refused to answer	128 (22.1)	23 (19.2)	105 (22.9)	
Afraid of ex-partner in the last year				>.99
Yes or unsure	5 (0.9)	1 (0.8)	4 (0.9)	
No	410 (70.9)	92 (76.7)	318 (69.4)	
Missing or refused to answer	163 (28.2)	27 (22.5)	136 (29.7)	
Risks per patient^h^				
0 to 4, n (%)	162 (28.0)	19 (15.8)	143 (31.2)	<.001
≥4, n (%)	416 (72.0)	101 (84.2)	315 (68.8)	
Mean (SD)	4.69 (1.92)	5.28 (1.84)	4.53 (1.92)	<.001

a Missing data and refusals to answer were included in the calculation, but the data are not shown in the table. Ethnicity, race, and spoken language under “Personal characteristics,” as well as health insurance under “Money and resources” in the PRAPARE tool overlap with the EHR data presented in Table 1. Therefore, these variables were not reported in this table; however, their distribution in the subsample was comparable to that of the full sample.

b Persistent poverty areas were defined as census tracts in which more than 20% of the population has lived below the poverty level for at least 30 years.

c Continuous and categorical variables were compared using a *t* test and chi-square test or Fisher exact test, respectively.

d Patients reported Family and Medical Leave Act and those who were disabled or retired were categorized as “Unemployed but not seeking work.”

e FPL: federal poverty level. The FPL was US $36,450 for a single individual in 2024.

f Resource needs included food, housing, transportation, childcare, utilities, phone, and others apart from medical or health care needs.

g High and medium-high stress included the response categories of “quite a bit,” “very much,” and “somewhat” stressed.

h Out of 22 negative events on the PRAPARE tool.

We collated the descriptive findings and created a summarization table to share with community stakeholders and academic investigators (Table 3). We linked our findings to a “lesson learned” goal that we then translated into a “callout” that was incorporated into our ongoing community engagement communications being used for LEAP study adaptation and implementation planning. For instance, we found that 64.1% (1714/2672) of the survivors in our sample had hypertension, leading to a callout that emphasized that survivors of cancer often experience other chronic conditions.

**Table 3.** Translation of electronic health record data into “lessons learned” shared with community stakeholders and academic investigators. These “lessons learned” reflect descriptive prevalence within the persistent poverty population and are intended for community engagement communication; they do not represent statistically significant differences between populations.

Social determinants of health for the patients with cancer in the health system living in persistent poverty^a^ census tracts	Goal for the “lesson learned” designed for lay public communication	“Lesson learned” related to survivors of cancer in the community shared to guide priorities for intervention adaptations
A total of 62% of patients with cancer living in persistent poverty census tracts are overweight or obese.	Raise awareness of the need for diet and exercise while avoiding a focus on weight alone.	Nearly 2 in 3 survivors of cancer in your community are at greater health risk due to excess body weight.
A total of 31% of patients with cancer have diabetes, and 64% have hypertension (41% have hyperlipidemia, and 20% have coronary artery disease).	Emphasize that cancer often occurs in the context of other medical conditions.	Cancer is not alone (2 out of 3 survivors of cancer may have another medical condition that healthy diet and exercise could help with).
A total of 55% of patients with cancer living in persistent poverty census tracts have Medicare, suggesting possible opportunities to leverage Medicare benefits related to nutrition and exercise counseling and programs.	Raise awareness of the need to help connect individuals with resources already available to them.	One in 2 survivors of cancer may have insurance coverage for diet and exercise education.
There are 25 different cancer types; prostate cancer was the second most prevalent cancer type in our study (17%), only slightly lower than breast cancer (21%).	Encourage people to think about a broad spectrum of cancer types and the importance of focusing on programs addressing the diverse needs of both male and female survivors of cancer.	Cancer has multiple faces in your community—men, women, and over 24 different cancer types.
The Social Vulnerability Index^b^ was high, indicating that the study areas were characterized by census-based indicators of low socioeconomic status.	Raise awareness of the major contributions to the Social Vulnerability Index for survivors of cancer specifically.	Survivors of cancer may face barriers due to older age, disability, discrimination, and limited financial resources.
Of the 2672 survivors of cancer with electronic health record data, 509 lived in persistent poverty areas.	The goal was to raise awareness of the prevalence of living in a persistent poverty area among health system patients.	One in 5 patients with cancer at the UAB^c^ Health System faces significant financial struggles.

a Persistent poverty areas were defined as census tracts in which more than 20% of the population has lived below the poverty level for at least 30 years.

b The Social Vulnerability Index (SVI) summarizes the extent to which a community is socially vulnerable to disaster. It was developed by the Centers for Disease Control and Prevention. SVI values range from 0 (least vulnerable) to 1 (most vulnerable).

c UAB: University of Alabama at Birmingham.

## Discussion

### Principal Findings

This study sought to characterize demographic, clinical, and SDOH factors among survivors of cancer in persistent poverty areas using EHR data to inform community-engaged adaptation of lifestyle modification interventions. We found that survivors of cancer in persistent poverty areas within our health system catchment were predominantly Black individuals and older adults with Medicare coverage and experienced high rates of obesity and chronic conditions, including hypertension and diabetes. Among those with social risk screening data, notable proportions reported transportation barriers, health care needs, food instability, fewer social interactions, and stress, consistent with the findings of a previous study [18].

Our data also highlight the burden of social risks in persistent poverty areas, consistent with previous research [2,8,19]. Health disparities among survivors of cancer are driven by a complex interplay of SDOH [20], which also serve as primary barriers to adequate health care access and physician communication [21-24]. Understanding these needs—as we did in this study—in planning tailored interventions will be imperative for sustainability and improved outcomes.

Survivors of cancer in persistent poverty areas often face multiple challenges of cancer survivorship, poverty, and clusters of problems that collectively affect their health. Adapting interventions to address the unique barriers that these populations face is important. In this study, we aggregated data on SDOH to guide the discussions with the planning, implementation, and evaluation working groups (community stakeholders and survivors and cosurvivors of cancer) convened as part of the ongoing LEAP study community engagement processes. This approach aligns with evidence-based cancer control strategies in persistent poverty areas [8]. Our findings support a comprehensive approach to promoting healthy eating and physical activity that addresses clusters of social risks rather than focusing on a single factor (eg, telehealth options to minimize transportation barriers, meal programs or home gardening to address food insecurity, and peer support groups to reduce social isolation).

The growing integration of SDOH screening into EHR systems represents a paradigm shift in how health care systems can support cancer control efforts in underserved communities [25]. Our approach aligns with emerging recommendations from national organizations emphasizing the importance of systematic SDOH data collection and documentation to inform care delivery and population health management [22]. The scoping review by Li et al [26] demonstrated that EHR-embedded SDOH data can be effectively linked to health outcomes and used to design targeted interventions, although standardization of data collection methods remains an ongoing challenge. Future efforts should focus on expanding SDOH screening beyond social work encounters to capture a more representative picture of social risks among survivors of cancer.

Community-based participatory research approaches have demonstrated effectiveness in optimizing health behaviors among survivors of cancer, particularly when interventions are tailored to local community resources and cultural contexts [3]. A recent systematic review found that most community-based interventions for survivors of cancer focused on physical activity and nutrition, with 88% showing improvement in at least one outcome measure; however, the review also highlighted a concerning lack of diversity in participant populations [27]. Our study provides foundational data to inform such community-based participatory research efforts by characterizing the specific demographic, clinical, and social risk profiles of survivors of cancer in our target communities—information essential for adopting evidence-based interventions to address local needs. The Active Living After Cancer program, for example, demonstrated that community-delivered physical activity interventions can successfully improve outcomes among medically underserved survivors of cancer and their caregivers when implemented through trusted community partnerships [28].

Notably, more than half (282/509, 55.4%) of the survivors of cancer in persistent poverty areas in our sample had Medicare coverage, suggesting opportunities to leverage existing Medicare benefits for lifestyle interventions. While Medicare currently covers medical nutrition therapy only for diabetes and chronic kidney disease, recent legislative efforts such as the Medical Nutrition Therapy Act for dietitian-delivered nutrition counseling will be important for this population [29]. In the meantime, health care systems serving persistent poverty areas can explore partnerships with community-based programs and leverage existing Medicare preventive services to address modifiable health behaviors among survivors of cancer.

The strengths of this study include using real-world health system data and NLP to capture both medical and nonmedical factors influencing health outcomes, enabling a more informed approach to addressing disparities prior to intervention development. Furthermore, the aggregated SDOH data were “translated” into a format for nonscience community-based partners to inform the ongoing community-engaged “co-design” of the planned LEAP intervention.

Limitations included the bias in the study sample of survivors of cancer, which was limited to the project target areas defined by low socioeconomic status indicators, and the PRAPARE- and NLP-derived data were also limited to individuals with the most acute social needs (eg, referral to social work). For this reason, we did not conduct formal hypothesis testing and did not focus on those results for interpretation in this report, and the findings may not be generalizable to other medical practice catchments. Our sample reflects survivors of cancer actively engaged in care at our health system, which may underrepresent patients with rapidly fatal cancers and those who have disengaged from care. This “survivor bias” should be considered when interpreting cancer type distributions; however, for the purpose of informing intervention adaptation, characterizing the population currently available for enrollment is appropriate.

Missing data were present for several variables, particularly PRAPARE (2094/2672, 78.4% missing) and NLP-derived SDOH (1076/2672, 40.3% missing) data, which reflects the clinical reality that these data are collected only for patients referred to social work services. This represents a form of data missing not at random, as patients with more acute social needs are more likely to have social work encounters. This limitation should be considered when interpreting prevalence estimates, which likely represent lower bounds for the broader population.

Despite these limitations, this is the first study to use an EHR-embedded social risk screener to provide insightful information for formative work among survivors of cancer in persistent poverty areas, offering valuable insights into their unique challenges and underscoring the importance of adapting interventions to address their specific social and health needs.

### Conclusions

This study demonstrates that leveraging SDOH data embedded in health system EHRs provides a feasible approach for characterizing populations of survivors of cancer in persistent poverty areas to inform community-based intervention adaptation. The social risk profiles identified highlight the importance of tailoring lifestyle interventions to address transportation, food access, and safety concerns alongside chronic disease management.

## Supplementary material

10.2196/81054 Multimedia Appendix 1 Natural language processing keywords used and social driver prevalence.

## Abbreviations:

EHR: electronic health record
LEAP: Leveraging Adaptation and Multilevel Implementation Strategies to Address Unique Health Promotion Challenges Among Cancer Survivors in Persistent Poverty Areas
NLP: natural language processing
PRAPARE: Protocol for Responding to and Assessing Patients' Assets, Risks, and Experiences
SDOH: social determinants of health
SIREN: Social Interventions Research and Evaluation Network
SVI: Social Vulnerability Index
