# Supplementary material for: Leveraging Innovative Electronic Health Record Data to Characterize Social Determinants of Health Among Survivors of Cancer in Persistent Poverty Areas: Cross-Sectional Study
Source: JMIR Cancer. 2026 Apr 13;12:e81054. doi: 10.2196/81054 (PMC13075633; doi:10.2196/81054)
Supplement: Multimedia Appendix 1 [file cancer-v12-e81054-s001.docx]

Supplementary Appendix

**Natural language processing of SDoH-related events**

We applied a rule-based natural language processing (NLP) approach to identify events related to unmet social determinants of health (SDoH) needs from both structured questionnaire items and free-text patient responses recorded in the electronic health record (EHR).

Text data were first preprocessed by tokenization, removal of stop words, punctuation, and non-informative characters, followed by normalization (e.g., lowercasing and stemming) to standardize text across sources. This preprocessing was applied consistently to both questionnaire prompts and patient responses.

We then conducted keyword-based text matching to identify SDoH-related events across five predefined domains: food insecurity, housing instability, transportation needs, interpersonal safety, and utility difficulties. Domain-specific keywords and phrases used to extract relevant events and identify unmet needs or negative responses are detailed in **Supplementary Table 1**. Keywords captured both the presence of SDoH-related topics (e.g., “housing,” “transportation,” “utilities”) and indicators of adversity or unmet need (e.g., “difficulty,” “lack,” “unable,” “unsafe”).

For structured yes/no questionnaire items, responses indicating an adverse condition were manually classified as indicating insecurity or unmet need according to the study protocol. For selected event types (e.g., “Barriers at Home” or “Home Bound Reasons”), any affirmative indication of a barrier was categorized as insecurity. Events related to suicidal ideation, substance use, or mental health were excluded from the interpersonal safety domain.

For each SDoH domain, patients were classified as experiencing unmet needs or insecurity if their most recent response indicated an adverse condition. Patients without such indicators were classified as having no identified unmet need (i.e., secure) for that domain.

Supplementary Table 1. List of keywords for searching events related to unmet needs for SDoH and negative events

| SDoH category | Total number of events | Text to extract events related to unmet needs | Text to identify unmet needs from responses | Note |
| --- | --- | --- | --- | --- |
| Food insecurity | 29 | enough, adequate, insecurity, running, worried, unable, malnutrition, access, availability, declined, needs | severe/moderate/mild, protein-calorie, malnutrition, often, sometimes, depletion, weight loss | If EVENT was a "Yes/No" question, categorized as "insecurity" by hand work. |
| Home instability | 33 | hazard, barriers, losing, worried, housing, homeless, safe, feel, appropriateness, adequate, needs, meet, pay, challenges | barrier identified, I do not have housing, not accessible, unstable | If patients reported any "Barriers At Home", categorized as "insecurity". If EVENT was a "Yes/No" question, categorized as "insecurity" by hand work. |
| Transportation needs | 48 | trans, lack, assistance, needs, med, strength, challenge, necessity, work, able, care, status, home, reasons, issues, drive, travel | trouble, unfamiliar, assistance, barrier, difficulty, transit, financial, lack | If patients reported any "Home Bound Reasons", categorized as "insecurity". If EVENT was a "Yes/No" question, categorized as "insecurity" by hand work. |
| Interpersonal safety | 254 | physical, emotion, safe, threat, afraid, concern, problem, harm, abuse, physical, partner, violence, violent, ex, attack, sex, domestic, hurt, force, pregnant | violence, unsafe, neglect, prisoner, financial, safe, difficulty, unsure | Excluded events regarding suicidal ideation, substance use, and mental health. If EVENT was a "Yes/No" question, categorized as "unsafe" by hand work. |
| Utility difficulties | 11 | service, utilit, strengths, electr, water, outlets, disconnected, gas | severe, moderate, often, sometimes | If EVENT was a "Yes/No" question, categorized as "insecurity" by hand work. |

SDoH: Social Determinants of Health.

Supplementary Table 2. Prevalence of social drivers of health according to new CMS 2024 requirements extracted using NLP.

| Variables | Overall | Persistent poverty areas | Non-persistent poverty areas | P-value |
| --- | --- | --- | --- | --- |
|  | (N=1594) | (N=302, 18.9%) | (N=1292, 454%) |  |
| Home instability |  |  |  |  |
| Insecure | 157 (9.8%) | 27 (8.9%) | 130 (10.1%) | 0.98 |
| Secure | 935 (58.7%) | 165 (54.6%) | 770 (59.6%) |  |
| Transportation needs | |  |  |  |
| Met | 1009 (63.3%) | 194 (64.2%) | 815 (63.1%) | 0.50 |
| Unmet | 243 (15.2%) | 52 (17.2%) | 191 (14.8%) |  |
| Food insecurity |  |  |  |  |
| Insecure | 200 (12.5%) | 41 (13.6%) | 159 (12.3%) | 0.07 |
| Secure | 658 (41.3%) | 97 (32.1%) | 561 (43.4%) |  |
| Interpersonal safety | |  |  |  |
| Safe | 374 (23.5%) | 71 (23.5%) | 303 (23.5%) | 0.17 |
| Unsafe | 30 (1.9%) | 10 (3.3%) | 20 (1.5%) |  |

CMS: Centers for Medicare & Medicaid Services; PP: persistent poverty. Note: Using the natural language process (NLP), an automated method for analyzing and interpreting text, allowed us to systematically identify relevant information from unstructured data. We first tokenized and normalized the text, and text matching to identify negative responses (e.g., difficulty, lack). Patients who were identified as having adverse events related to any of the items were categorized as having unmet needs or experiencing insecurity (e.g., insecurity vs. security). Categorical variables were compared using the Chi-square test or Fisher’s Exact test. Missing data: home instability (n=459, 29.6%), transportation needs (n=299, 19.3%), food insecurity (n=693, 44.7%), interpersonal safety (n=408, 26.3%). Data for utility difficulties were not shown as 98% (n=1,562) of patients had missing data.
